# Comparison of Mechanical Properties of Hemp-Fibre Biocomposites Fabricated with Biobased and Regular Epoxy Resins

**DOI:** 10.3390/ma13245720

**Published:** 2020-12-15

**Authors:** Vicente Colomer-Romero, Dante Rogiest, Juan Antonio García-Manrique, Jose Enrique Crespo

**Affiliations:** 1 Instituto de Diseño para la Fabricación (IDF), Universitat Politecnica de Valencia, Camino de Vera s/n, 46022 Valencia, Spain; vicoro@mcm.upv.es (V.C.-R.); dante.rogiest@gmail.com (D.R.); 2 Instituto de Tecnología de Materiales, Universitat Politecnica de Valencia, Plaza de Ferrándiz y Carbonell, 2, 03801 Alcoy, Spain; jocream@dimm.upv.es

**Keywords:** green composites, natural fibres, hemp fibres, bending test, tensile test, biocomposite

## Abstract

Bio- and green composites are mainly used in non-structural automotive elements like interior panels and vehicle underpanels. Currently, the use of biocomposites as a worthy alternative to glass fibre-reinforced plastics (GFRPs) in structural applications still needs to be fully evaluated. In the current study, the development of a suited biocomposites started with a thorough review of the available raw materials, including both reinforcement fibres and matrix materials. Based on its specific properties, hemp appeared to be a very suitable fibre. A similar analysis was conducted for the commercially available biobased matrix materials. Greenpoxy 55 (with a biocontent of 55%) and Super Sap 100 (with a biocontent of 37%) were selected and compared with a standard epoxy resin. Tensile and three-point bending tests were conducted to characterise the hemp-based biocomposite.

## 1. Introduction

Composite materials have gradually found applications in several industrial branches. The advantages of the composites are undeniable, and range from lower cost over a lower specific weight to higher impact resistances [1]. Nowadays, other properties, such as recyclability and CO_2_ balance, play an important role in the selection of the material, and this is exactly where the synthetic composites are lagging behind [2,3]. In their quest for ever more fuel-efficient vehicles, the designers in the automotive sector are always looking for the highest structural efficiency at the lowest possible weight. Such an objective has led to the extensive use of composite materials in a wide variety of areas. At present, the relatively new bio- and green composites, which are (partially) based on biomaterials, are highly popular. These biobased composites have huge advantages in terms of environmental impact, handling, and recycling over the synthetic composite materials. On the downside, they do not offer the same mechanical performance as synthetic composites and, therefore, their use is mostly limited to non-structural applications.

Natural fibre composites (NFCs) consist of a traditional polymer as a matrix material, reinforced with natural fibres. Notably, an NFC becomes a green composite when it contains a biobased matrix material. In 2010, the total annual European composite production volume equaled about 2.4 million tonnes [4]. The annual production volume of natural fibres in Europe in the same year equaled about 315,000 tonnes, and, owing to their promising properties, the European production volume of natural fibre-based composites is likely to more than double by 2020 [4]. Of all industries, the automotive sector is probably the biggest promoter, user, and developer of green composites [3,5,6,7]. Above all, the automotive sector is continuously trying to improve the fuel efficiency of vehicles. Although considerable work is being done on improving the efficiency of the vehicle drivetrain (engine, transmission, etc.), weight has become an important factor (like in the aerospace industry), as vehicles are becoming increasingly heavy. This weight increase is mostly due to the continuous increase in the vehicle size and the steady rise of comfort and safety equipment on board. To meet the emission standards issued by the European Union (EU), this trend must be stopped, and preferably reversed, using lightweight materials during the production of the vehicle chassis and body. The need to save weight in the current vehicle design leads to an increase in the use of aluminium and high specific strength steel alloys for vehicle chassis production. Currently, many of the interior panels comprise NFCs. Door linings, boot linings, seat backs, headliner, parcel shelves, and vehicle under floors are only a few examples of parts that are already produced using NFCs. For new hybrid or full electric vehicles, weight saving is even more important, as the existing battery technology used in these types of vehicles further increases their weight, leading to undesirably small driving ranges. Therefore, lightweight composite materials should preferably replace aluminium and high specific strength steels. This shift, however, is not as easy as it seems; the EU end-of-life vehicle directive states that, from 2015, 85% of the mass of each new vehicle has to be recyclable [7,8,9]. This recyclability is exactly the Achilles’ heel of traditional composite materials. A possible solution has been implemented by BMW, who uses full carbon fibre reinforced with a thermosetting matrix (Araldite LY 3585 epoxy with Hardener XB 3458) for the chassis of their i3 and i8 vehicles.

The objective of this investigation is the development of a biocomposite consisting of hemp fibres and a biobased epoxy matrix material. The different matrix materials in combination with the hemp fibres are compared to evaluate their future potential application in the automotive industry.

## 2. Materials and Methods

In this work, two types of hemp fibre plain weaves of 320 and 470 g/m^2^ were used. The mechanical properties of this fibre are:


**Tensile Strength (Mpa)**

**Density (g/cm^3^)**

**Specific Strength (MNm/kg)**

**E-Modulus (GPa)**

**Strain (%)**
550–9001.50.6701.6

These fibres were combined with three types of resin matrix materials, each of them with fossil fuel to a different extent; specifically, Greenpoxy 55 resin (GP 55, Sicomin, Chateauneuf les Martigues, France) and biobased epoxy resin system Super Sap 100/1000 (SS 100/1000, Entropy Resins, Hayward, CA, USA) with a biocontent of 55% and 37%, respectively, were adopted; as a reference, a third classical epoxy resin system, SP 110 epoxy resin (SP 110, Gurit, Zürich, Switzerland), was used.

Liquid composite molding is, in many ways, the most suitable composite production process for this type of application [10]. In addition, the vacuum infusion process was used for producing the green composite test specimen. The composite testing standards ISO 14125 and ASTM d3039 [11,12] prescribe a test specimen thickness of at least 2 mm; therefore, the number of layers in the laminate varies with the used fibre type. The tested laminates consisted of three layers for the 470 g/m^2^ sample, and of five layers for the 320 g/m^2^ reinforcement. After carefully reviewing the technical datasheets of each individual resin [13,14,15], post-curing treatment was conducted at room temperature for a period of 14 days (or less, depending on the curing specifications). This curing process precedes the abrasive water jet cutting of the test specimen.

The tensile testing standard for fibre-reinforced plastics is the ASTM D3039/D 3039M–08 [12] standard. As all produced plates are balanced and symmetric laminates, the recommended specimen dimensions are 250 mm × 25 mm (L × W) with a thickness of 2.5 mm. Notably, these are the recommended dimensions, and therefore they are not binding. The use of tabs (local reinforcements of the specimen in the gripping area) is not mandatory for this specific type of composite.

The standard for a three-point bending test of fibre-reinforced materials is ISO 14125:1998 [11]. According to this standard, the green composite material produced here is a class III material. This results in recommended specimens with a length of 60 mm, a width of 15 mm, and a thickness of 2 mm. Plates were fabricated (460 mm × 420 mm) with a thickness of at least 2 mm. From each test specimen, five were cut out of the plate, which is the minimum number of samples to analyse to obtain statistically viable data, according to all applicable testing standards [11,12,16,17]. For further investigation of the 90 green composites, considering the sheer number of specimens to be cut and the importance of their dimensional equality, all specimens were cut using a computer numerically controlled abrasive water jet cutting machine.

**Figure 1:**
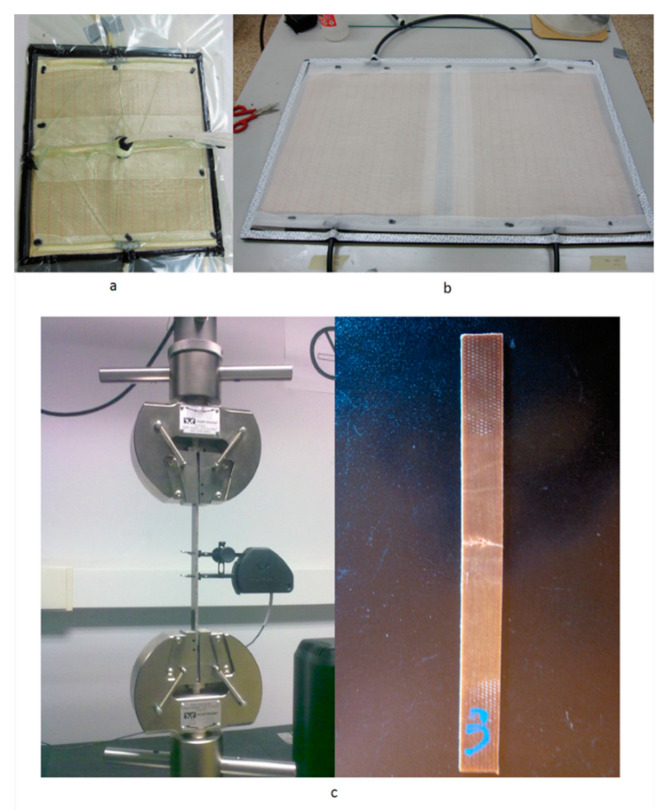
(**a**) Resin Transfer Molding (RTM) process; (**b**) production preparation; (**c**) tensile testing.

All tensile tests were performed on an Instron© (Instron, Norwood, MA, USA) 5967 tabletop test system (see Figure 1c). For constant head-speed testing, the machine was set to a standard head displacement velocity of 2 mm/min, which conforms to the standard for tensile testing ASTM D3039/D 3039M—08 [12]. The distance between the grips, the gauge length (Lg), is set to 140.9 mm as a standard for all tensile tests.

For each test, the software was configured to generate the following output:

- Testing time (*t*) (s);
- Tensile force (F_t_) carried by the test specimen (N);
- Displacement (*δ*) measured with the extensometer (mm);
- Tensile modulus of elasticity (E) in the test direction (MPa).

The output data were used to calculate the exact properties of each tested composite type. Before starting the actual testing, each specimen was individually characterised by determining its size and weight. During testing, the specifications of each test specimen were input into the Bluehill^©^ software.

**Figure 2:**
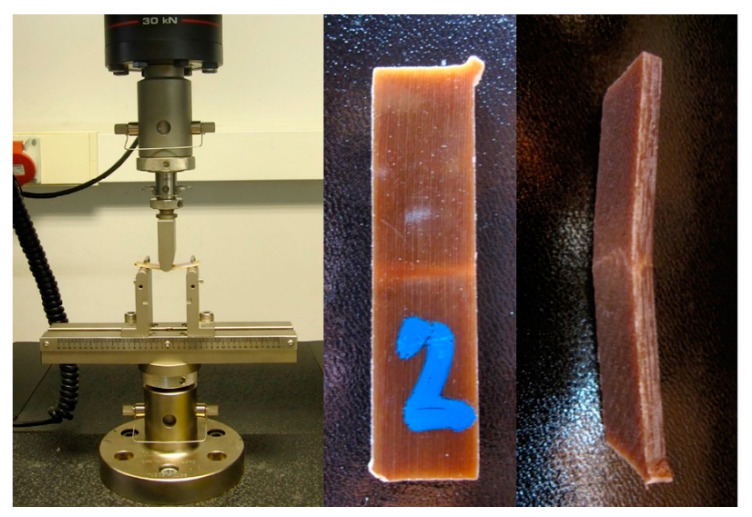
Three-point bending test.

After the tensile test, the testing rig was equipped with special tooling to conduct a three-point bending test (see Figure 2), as prescribed by the testing standard [11]. The constant deformation velocity (*v*) used to perform the test was calculated using Equation (1).

(1) $$v=\frac{\varepsilon^{\prime }L^{2}}{6h}$$

The distance between the supports (*L*) was set to be 45 mm for all tests, and the bending velocity (*ε*′) was 1% min. Consequently, the deformation velocity depended only on the test specimen thickness (*h*), giving a value of approximately 1.69 mm/min. The testing software was configured to generate the following output:

- Testing time (*t*) (s);
- Bending force (F_b_) carried by the test specimen (N)—bending deformation (s) (mm);
- Bending modulus of elasticity (E_b_) (MPa).

Some general properties of the composite types were calculated (see Table 1). In this specific case, the calcination method cannot be applied to determine the fibre volume content, as the hemp fibres used here are not heat resistant. The fibre volume fraction fluctuates, depending on both the used fibres and matrix materials. This clearly influences the relative performance of each composite type. The fibre volume fraction of a composite is normally obtained by applying the so-called calcination method (a method prescribed by ISO standard 1172). Using this method, the polymer matrix material is burned off, whereafter the remaining reinforcement fibre fraction is weighed. These data can then be used to calculate the fibre volume fraction of the original composite. It is impossible to use the calcination method in this specific case, because of the simple fact that the hemp fibres used here are not heat resistant. Therefore, the fibre volume fraction is calculated using the following equation:

(2) $$V_{f}=V_{c}-\left(\frac{M_{c}-M_{f}}{\rho_{m}}\right)$$

where *V_f_* is the volume fraction, *M_c_* is the mass of composite, *M_f_* is the mass of reinforcement, and *ρ_m_* is the matrix density.

## 3. Results and Discussion

Some production difficulties were encountered during the vacuum infusion of the testing plates, mostly due to the high viscosity of the biobased epoxy resins. However, after production, several tests were conducted on the hemp fibre composites and some interesting conclusions were drawn.

The higher viscosity of the biobased epoxy resins (approx. 0.6 Pas) caused some difficulties during the vacuum infusion. The epoxy resins used for this investigation were heated to a certain temperature to lower their viscosity, which, on the downside, caused a decrease in their pot life. Finding an equilibrium between both requirements is important for a trouble-free infusion process. The viscosity-dictated behavior was far less problematic in the Greenpoxy 55 than in the Super Sap 100 epoxy resin. Before starting production, the impact of the polymerisation retardants or substances, used to lower the resin viscosity, on the performance of the composite needs to be investigated. It is shown that biobased epoxy resins are more complex materials in terms of processing, but if a correct impregnation is carried out, the results obtained are affected in the same way as the fossil fuel resins.

All tensile and three-point bending tests were conducted according to their respective testing standards. The evaluation of the mechanical properties of the biocomposites, resulting from the tensile and three-point bending tests, can be found in Table 2 and Table 3, respectively. It can be seen that the tensile and flexural behavior of the plates made with Greenpoxy 55 and Gurit SP 110 have a similar behavior, with the properties of Super Sap 100 lowered by 20% in strength for tensile and bending properties. The performance of the SP resin is better in all cases, however, it is observed that its replacement by bioresins may be feasible for certain applications. These mechanical properties can be improved through an adequate fibre pretreatment to increase compatibility without compromising the fibre strength.

**Figure 3:**
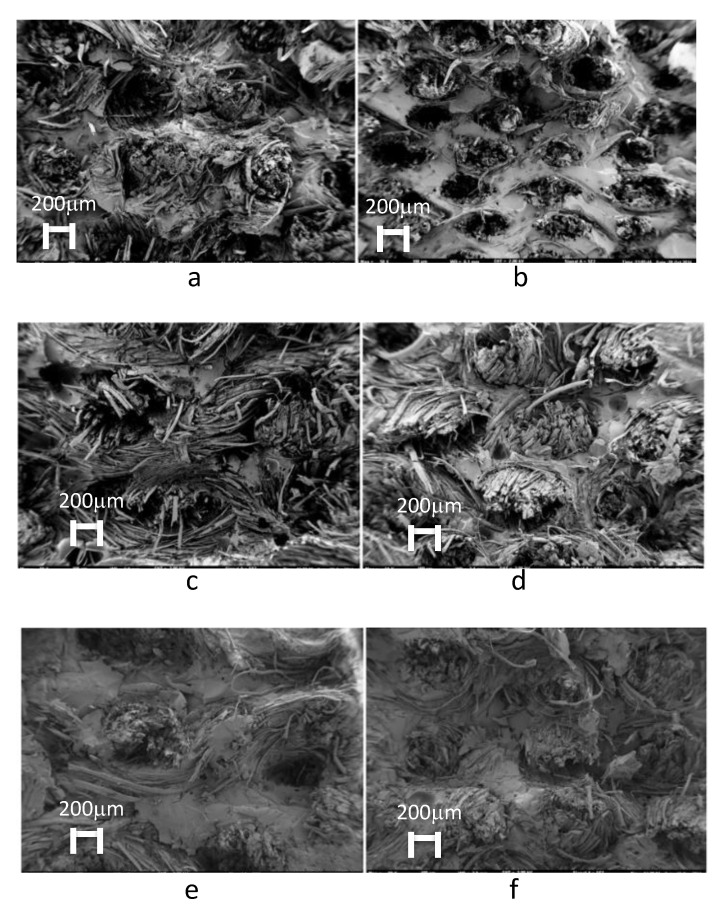
Scanning electron microscopy images of the tensile specimen fractures: (**a**) #1, (**b**) #2, (**c**) #9, (**d**) #10, (**e**) #17, (**f**) #18.

Detailed scanning electron microscopy (SEM) images of the tensile fractures of each specimen were analysed. The SEM images, in Figure 3, show that the fibre–matrix bond between the fibres and the biobased epoxy resins (see Figure 3,b,e,f) is weaker than that observed in the specimens fabricated using the traditional epoxy resin (Figure 3c,d). The gaps between the fibre and matrix of some of the images (Figure 3e,f) are different than those observed in traditional resin (Figure 3c,d), and this has allowed for obtaining similar values to Greenpoxy 55 and Gurit SP110 (Figure 3c–f). In the case of Super Sap 110 (Figure 3a,b), it is observed that the fibre separates from the matrix, leaving the resistance to the fibre, so it is observed that the fibre is torn away and does not section with the matrix as in the other images.

## 4. Conclusions

As recyclability and CO_2_ emissions become increasingly more important, hemp fibre-reinforced composites can be used as mechanically resistant components in a variety of applications. To further characterise the developed biocomposites, additional mechanical tests, such as compression, interlaminar, and in-plane shear tests, should be conducted. In particular, when analysing the bending stiffness results, a significant difference between the bio-epoxy and standard epoxy resins can be observed, a phenomenon also reported by Madhu et al. [17]. This difference results from variations in the fibre–matrix adhesion, as natural fibres generally tend to cause far more problems (when untreated) than glass or carbon fibres. The variation of the mechanical properties, which depend on a large number of factors during cultivation, is a crucial problem for natural products (like hemp fibres). According with the traction and bending results obtained and the SEM images, we can conclude that the resistance of composites made with bioresins has a minimum reduction of 20%, allowing the substitution of traditional resins for those based on biomaterials in the same applications, for example, small wind blades, car monocoques, etc.

## Figures and Tables

**Table 1:** General properties of hemp fibre-reinforced plastic.

Composite Plate Number	Fibre Type	Number of Layers (nl)	Composite Density, ρ	Fibre Volume Fraction
**Super Sap 100**				
1	470 (+/−90)	3	1231.18	36.56
2	320 (+/−90)	5	1250.63	37.59
**Epoxy SP 110**				
9	470 (+/−90)	3	1224.86	43.35
10	320 (+/−90)	5	1254.85	44.23
**Greenpoxy 55**				
17	470 (+/−90)	3	1246.98	35.06
18	320 (+/−90)	5	1235.14	44.86

**Table 2:** Tensile properties of hemp fibre composites.

Composite Plate	Strength (MPa); σ_u_	Std. Dev. (MPa) S_n−1_	Strain (%): ξ_u_	Modulus (MPa)	Specific Strength (kNm/kg); σ_s_	Specific Modulus (kNm/kg); E_s_
**Super Sap 100**						
1	39.82	1.45	4.14	4558	32.34	3702.14
2	47.9	0.91	10.6	3782	38.30	3022.48
**Epoxy SP 110**						
9	49.18	0.96	2.48	6410	40.15	5233.27
10	53.92	0.65	3.40	6410	42.97	5108.20
**Greenpoxy 55**						
17	49.68	1.28	2.63	4570	39.84	3664.87
18	48.22	1.05	4.91	5860	39.04	4744.40

**Table 3:** Bending properties of hemp fibre composites.

Composite Plate	Strength (MPa); σ_u_	Std. Dev. (MPa); S_n−1_	Strain (%): ξ_u_	Modulus (MPa)	Specific Strength (kNm/kg); σ_s_	Specific Modulus (kNm/kg); E_s_
**Super Sap 100**						
1	79.60	3.34	3.98	3960.00	64.65	3216.43
2	79.72	1.97	6.98	3110.00	63.74	2846.75
**Epoxy SP 110**						
9	100.82	6.06	3.18	5292.00	82.31	4320.51
10	113.00	1.58	3.66	5838.00	90.05	4652.37
**Greenpoxy 55**						
17	92.72	3.02	3.5	4446.00	74.36	3565.43
18	100.26	3.28	4.26	4912.00	81.17	3976.88
